# How to find genomic regions relevant for gene regulation

**DOI:** 10.1515/medgen-2021-2074

**Published:** 2021-08-14

**Authors:** Xuanzong Guo, Uwe Ohler, Ferah Yildirim

**Affiliations:** Department of Psychiatry and Psychotherapy, Charité–Universitätsmedizin Berlin, Corporate Member of Freie Universität Berlin and Humboldt-Universität zu Berlin, 10117 Berlin, Germany; Max-Delbrück-Center for Molecular Medicine in the Helmholtz Association (MDC), Berlin Institute for Medical Systems Biology, 10115 Berlin, Germany; Department of Biology, Humboldt-Universität zu Berlin, Berlin, Germany

**Keywords:** non-coding genome, *cis*-regulatory regions, transcriptional regulation, epigenetics, transcription factor binding

## Abstract

Genetic variants associated with human diseases are often located outside the protein coding regions of the genome. Identification and functional characterization of the regulatory elements in the non-coding genome is therefore of crucial importance for understanding the consequences of genetic variation and the mechanisms of disease. The past decade has seen rapid progress in high-throughput analysis and mapping of chromatin accessibility, looping, structure, and occupancy by transcription factors, as well as epigenetic modifications, all of which contribute to the proper execution of regulatory functions in the non-coding genome. Here, we review the current technologies for the definition and functional validation of non-coding regulatory regions in the genome.

## Introduction

Completion of the human genome sequence [1], which contains roughly 3.3 billion nucleotides, quickly led to the recognition that only about 2 % of the human genome is protein coding, while the remaining nearly 98 % do not code for proteins. Functional high-throughput studies have indicated that a considerable fraction of non-coding sequences, with estimates on the order of 5–10 %, harbor key functional elements responsible for the regulation of complex temporal and tissue-specific gene expression in different cell types of the human body [2]. Genome-wide association studies (GWAS) have mapped numerous loci that are associated with complex phenotypic traits. More than 90 % of these GWAS variants are located in non-coding regions of the genome clustering in and around regulatory elements. How these variants act on the phenotype has thus fueled new interest into the functional interaction between these quantitative trait loci (QTLs) and their associated genes [3].

With the genome sequence available, the next pressing challenges were to annotate all gene regulatory regions and understand their function. Due to the rapid progress in DNA sequencing technologies over the course of the last decade, combined with the development of novel *in silico* analysis and modeling tools, our understanding of the non-coding human genome has finally advanced to the point where we can begin to address these challenges.

**Figure 1:**
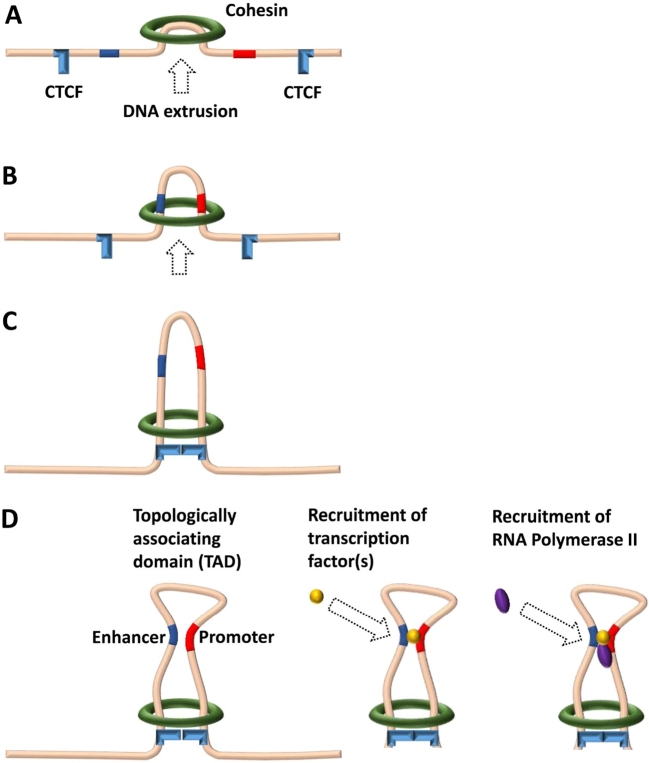
*Model of promoter–enhancer interaction in a chromatin loop extruded by the cohesin complex.* **(A and B)** The ring-shaped ATPase cohesin complex initiates the extrusion of the chromatin fiber and keeps translocating along the chromatin, forming a DNA loop in the process. **(C)** Translocating cohesin stops at a pair of convergent CTCF sites that form the boundary of the chromatin loop or the TAD. The CTCF sites have to be in a proper orientation to each other in order to stop cohesin. **(D)** Enhancers and promoters within a single loop are prone to interaction due to proximity, resulting in the recruitment of transcription factor(s) and of RNA polymerase II, thereby initiating gene transcription.

Transcriptional regulatory regions, also referred to as *cis*-regulatory regions or modules, are often grouped into promoters, enhancers, and insulators. Promoters are proximal regions around the transcription start sites (TSSs) of genes which contain DNA elements that recruit the basal transcriptional factors (TFs) and, ultimately, **RNA P**olymerase II (RNAP II), setting in motion the transcription machinery. Enhancers are distal *cis*-regulatory regions that recruit sequence-specific TFs along with chromatin remodeling co-factors, thereby exerting an influence on the transcription initiation at the promotor. A prominent feature of enhancers is their key role in tissue-specific gene expression. Highly regulated genes can be influenced by up to several dozen enhancers [4], [5]. Their coordinated activity with promoters dictates the dynamic temporal and spatial regulation of transcriptional programs in different cell types.

To this end, proximal and distal regions contain several short sequence elements called TF binding sites (TFBSs) that attract specific TFs (see Leitz et al., this issue). Distal regions with an effect on gene transcription can be located downstream or upstream of the promoter, as well as inside the gene or at a large distance ranging from hundreds to tens of thousands of nucleotides. Finally, insulators act as barriers to prevent the spread of heterochromatin and to block distal regions from acting on unrelated promoters. In vertebrate cells, this enhancer-restrictive function of insulators is mediated by binding of the CCCTC-binding factor (CTCF), which plays a crucial role in organizing the genome into topologically associating domains (TADs) [6]. Currently, the generation of TADs is best explained by the loop extrusion model (Figure 1A–C), where the ring-shaped cohesin complex pinches a loop out of the chromatin fiber and continues to propel chromatin *via* its ATPase activity until it reaches a pair of convergent CTCF sites. Promoters and enhancers within a loop are prone to interaction due to proximity (Figure 1D). Notably, while enhancers are the primary drivers for cell type-specific gene expression programs, insulator positions have largely been found to be similar in different cell types.

Regulatory regions of the genome are marked by epigenetic signals, which comprise DNA methylation and chemical modifications of the histones. Eukaryotic DNA is tightly packed into chromatin by means of nucleosomes, consisting of 147 bp of DNA wrapped around a histone octamer, separated by stretches of linker DNA. Modification of these histones by means of methylation or acetylation has an influence on the packing density and accessibility of the chromatin. Unlike the underlying static DNA sequence, these modifications can dynamically respond to and alter the cellular states. Specific epigenetic modifications and their particular combinations, the so-called “histone code” [7], are associated with distinct genomic regulatory features and their activity states [8], providing key spatial and temporal functional information.

In this review, we focus on genome-wide approaches for defining genomic regulatory regions, including high-throughput profiling of open chromatin, epigenomic marks, and long-range promoter–enhancer interactions. We also discuss advances in the genome and epigenome editing assays for functional analyses of the identified candidate regulatory regions, enabling their validation for potential causal roles in target gene regulation.

## Annotation of candidate regions

### Accessible chromatin: From DNase- to ATAC-seq

In addition to being the structural core unit of chromatin, nucleosomes and their positioning throughout the genome have pivotal functions for regulating the accessibility of binding sites to TFs and the basal transcriptional machinery. During transcriptional activation, binding of TFs, orchestrated by the action of histone remodelers, results in the destabilization of nucleosomes at *cis*-regulatory regions. Accessible regions of the genome are therefore the primary genomic candidates for harboring regulatory elements and have been historically characterized based on their hypersensitivity to DNase I digestion [9].

DNase I hypersensitivity forms the basis of DNase I-hypersensitive sites sequencing (DNase-seq), a method for the genome-wide and high-throughput identification of DNase I-hypersensitive sites [10]. DNase-seq has become a standard technique for probing chromatin accessibility and was extensively used by the ENCODE [11] and Epigenomics Roadmap [12] consortia to study cell-specific chromatin accessibility and its relation to gene expression in numerous cell and tissue types.

Since its introduction in 2013, the assay for transposase-accessible chromatin using sequencing (ATAC-seq) has increasingly replaced DNase-seq as a fundamental tool for genome-wide mapping of open chromatin regions [13]. ATAC-seq uses a genetically engineered hyperactive Tn5 transposase that is capable of inserting DNA sequencing adapters specifically into regions of open chromatin. This allows targeted PCR amplification of open chromatin fragments, followed by subsequent construction of a next-generation sequencing (NGS) library, which represents the entirety of open chromatin. ATAC-seq has risen in popularity due to its simple and time-efficient protocol and substantially lower amount of required starting material, ranging from 500 to 50,000 cells, while generating data with comparable sensitivity and specificity as DNase-seq. Because the ATAC-seq protocol does not involve any size selection steps, it can simultaneously identify nucleosome positions and accessible regions. As ATAC-seq can work with little source material, it is an ideal tool for projects with limited sample availability, such as investigation of differentiated cells derived from induced pluripotent stem cells (iPSCs) and patient specimens.

The binding of regulatory factors within accessible regions leaves a so-called “footprint,” i. e., a region of the DNA that is occupied by the TF and thus prevents DNase I cleavage or Tn5 insertion. DNase-seq and ATAC-seq have therefore also been used to study TF occupancy genome-wide at nucleotide resolution by “TF footprinting” [14] (Leiz et al., this issue). The reliability of both DNase- and ATAC-seq for this purpose is influenced by sequence cleavage biases of the enzymes [15], meaning that they are not always applicable and that success will depend on the condition and type of TF [16].

### Activity of candidate regions

**Figure 2:**
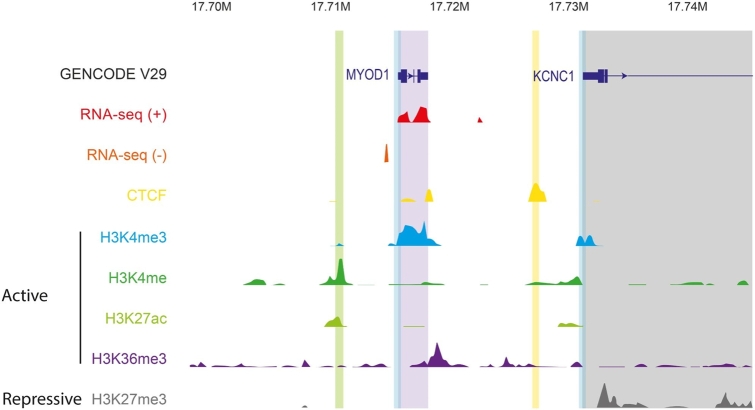
*Schematic depiction of the browser view of the human MYOD1 locus, overlaid with RNA-seq, CTCF ChIP-seq, and various histone ChIP-seq tracks.* Sequencing data were generated from human myotubes in culture by the ENCODE Consortium. For the gene annotation track at the top, blue indicates protein coding genes, thick bars protein coding regions (exons), narrow bars untranslated regions (UTRs), lines introns, and arrows the direction of transcription. RNA-seq signals of plus and minus strands are shown, as are the most common and robust active and repressive histone marks. The positions of gene regulatory regions are depicted by vertical shades: promoters (blue), enhancers (green), TAD boundaries (yellow), actively transcribed gene bodies (purple), and repressed gene bodies (gray). In this example the gene *MYOD1* is transcribed, while *KCNC1* is repressed.

DNase- and ATAC-seq assays reveal accessible genomic loci, but they do not distinguish between different kinds of regulatory regions and their activity, i. e., if they are engaged in regulating a gene in a particular context. The regulatory state of a region is however reflected in patterns of histone modifications. DNA is wrapped around a nucleosome core, which is a tetramer composed of different histone proteins with tails that can be modified, most frequently by methylation or acetylation. These modifications serve as road signs for gene regulation. Using **Ch**romatin **I**mmuno**P**recipitation (ChIP), Hebbes et al. (1988) first established a direct link between core histone acetylation and transcriptionally active chromatin [17]. As more and more types of histone modifications were discovered, scientists put forward the existence of a “histone code,” in contrast to the “genetic code,” which orchestrates the transcriptional program of the invariant genome. Table 1 lists the best-known and most robust histone marks. Due to the specificity of antibody–antigen recognition, ChIP has been successfully adapted to the genome-wide profiling of histone modifications, histone variants, and DNA methylation. This method is called ChIP sequencing (ChIP-seq), where all the DNA fragments are sequenced that are precipitated with the protein of interest, e. g., a transcription factor or a modified histone. This allows bioinformatics to remap the position in the genome from where the respective chromatin fragments were precipitated. Figure 2 shows a schematic genome browser view of the human *MYOD1* locus with RNA sequencing (RNA-seq) tracks of plus and minus strands and ChIP-seq tracks of CTCF, H3K4me, H3K4me3, H3K27ac, H3K27me3, and H3K36me3 generated by the ENCODE Consortium from a human myotube culture derived from a skeletal muscle myoblast line.

**Table 1:** *Examples for frequent histone marks*. The table lists the names of five common histone marks, their preferred genomic locations, and their relations with respect to gene transcriptional activity; e. g., H3K4me3 = trimethylation at the fourth lysine residue of the histone H3 protein; H3K27ac = acetylation at the 27th lysine residue of the histone H3 protein.

Histone mark	Location	Activity
H3K4me3	around the TSS	active
H3K4me1/me2	at the enhancer	active or primed
H3K27ac	at the enhancer	active
H3K36me3	within the gene body	active
H3K27me3	at the promoter and within the gene body	repressive

DNA methylation has long been associated with gene repression, but research over the course of the past decades has shown that DNA methylation participates in multiple cellular functions, some of which are still not fully understood. These include repression of transposons, inactivation of the X-chromosome, and genomic imprinting. Most DNA methylation happens at CpG dinucleotides. This short palindromic sequence instructs the methylation of daughter strands during DNA replication.

Methylation is classically investigated by bisulfite sequencing [18]. Treatment with bisulfite before sequencing converts cytosine residues to uracil with the exception of 5-methyl-cytosines. Therefore, only methylated cytosines are retained. The computational comparison of sequence reads from treated and untreated DNA samples allows the exact determination of CpG positions at single-nucleotide resolution. NGS allows genome-wide analysis of methylation patterns, a method called Bis-seq [19].

The low overall percentage of CpG dinucleotides in the mammalian genome stands in contrast to the high percentage found at CpG islands (CGIs), where CpG dinucleotides cluster in genomic regions of about 1 kb. Over two thirds of mammalian promoters, including almost all housekeeping genes and several developmental genes, coincide with CGIs. CGI promoters are rarely methylated; in these instances, gene silencing is achieved by H3K27 methylation [20]. While methylation of some TFBSs deters binding, many TFs in the extended homeodomain family did prefer methylated CpG in an *in vitro* assay [21]. Interestingly, the mammalian genome rewrites its methylation pattern twice during development, once after fertilization and once after germline specification.

**Figure 3:**
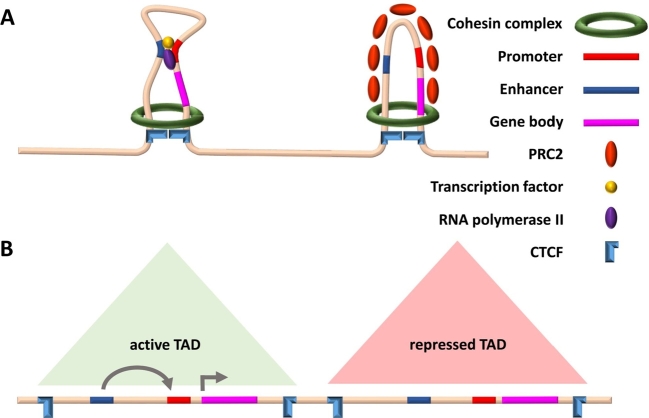
*Chromatin conformation capture methods profile long-range interactions between regulatory sequences, thereby identifying TADs.* **(A)** TADs can be repressed by binding of chromatin remodelers such as Polycomb Repressive Complex 2 (PRC2), which compacts the regions and prevents transcription. **(B)** Depicted are two TADs, one active (green) and one repressed (red). In the active TAD, a long-range promoter–enhancer interaction leads to active transcription. In the repressed TAD, silencing histone marks such as H3K27me3 recruit epigenetic modifiers and chromatin remodelers such as PRC2 to compact chromatin, preventing transcription. TADs are identified by directional ligation events in the 3C-based assays.

Through ENCODE and other concerted efforts to systematically profile the epigenomic landscapes in human cell lines, primary cell types, and model systems, it became obvious that the number of possible combinations of histone combinations and other epigenetic states at genomic loci exceeded the capabilities of manual human exploration. This is therefore one area where computational analyses, and in particular the use of machine learning, has made a crucial impact. In 2010, Ernst and Kellis identified 51 “chromatin states” *de novo* with a simple combinatorial model of 38 histone modifications and other epigenetic information. These states fit with existing genomic annotations [8], although subsequent publications have proposed a less complex picture with fewer states [22], [23].

### Regions in their spatial context

While distal regulatory regions can be confidently identified using chromatin accessibility analysis and ChIP-seq for epigenetic marks, it is challenging to link them to their target genes by this information alone. Distal regions can be located far from their target promoters, upstream or downstream, exerting their function on possibly multiple targets over large genomic distances. Advances in genome-wide chromatin interaction profiling demonstrated that many regulatory regions that are distal on the linear genome come into close physical proximity as a result of chromatin looping and higher-order organization of the 3D structure of the chromatin (Figure 3A). Among these technologies, Hi-C, a method based on chromatin conformation capture (3C), and **Ch**romatin **I**nteraction **A**nalysis with **P**aired-**E**nd **T**ags (ChIA-PET) are two state-of-the-art genome-wide assays for studying chromatin interactions based on nuclear proximity ligation that enable the detection of genomic regulatory regions brought into close spatial proximity by long-range chromatin looping.

Chromatin conformation capture methods investigate the interaction between two loci (3C), between one locus and the rest of the genome (4C), or between multiple loci (5C). Hi-C is based on proximity ligation of cross-linked DNA via a biotin linker, which is also used to pull down the DNA fragments, followed by high-throughput sequencing [24]. Unlike previous approaches, Hi-C can, at least in theory, capture all genome-wide chromatin interactions. Hi-C studies revealed that the human genome is organized into large TADs, where distal–proximal regulatory region interactions are facilitated within the same TAD [25]. CTCF and the cohesion complex play key roles in establishing and maintaining TAD boundaries, the positions of which are invariant in different cell types and whose disruption can result in aberrant long-range distal–proximal regulatory region interactions (Krude et al., this edition), leading to dysregulation of target genes in disease conditions [26], [27]. TADs as a whole can be repressed by binding of chromatin remodelers, such as Polycomb Repressive Complex 2 (PRC2), which compacts the chromatin and inhibits enhancer–promoter interactions (Figure 3B). While there is a body of evidence supporting the concept of preferential intra-TAD promoter–enhancer contacts, recent studies identified significant promoter interactions with distal regulatory sites that crossed TAD boundaries, indicating that TAD boundaries can be overcome at certain incidences [28], [29].

A main limitation of using Hi-C methods to study distal–proximal regulatory region interactions is the extremely high number of sequencing reads required to achieve a resolution necessary to resolve individual interactions. Billions of reads are needed to reach the current highest resolution of 5–10 kb. These limitations can be overcome by Capture Hi-C (CHi-C), which enables interaction analysis of targeted regulatory regions by manageable amounts of sequencing data [30]. Alternatively, ChIA-PET enriches for interactions associated with a particular chromatin modification or TF. The method can create high-resolution (< 1 kb) maps of chromatin interactions mediated by a protein of interest, thereby linking distal TFBSs to their target genes [31].

## Validating candidate variants

The “genetic code” for translation of nucleotide triplets into amino acids has been known for decades. While the impact of mutations on the protein coding function can be linked to large numbers of clinical disease phenotypes, there appears to be no straightforward “regulation code” counterpart. Many enhancers can influence one gene, one enhancer can affect several genes, and their joint activity is encoded in the combination of functional sequence elements, their redundancy, and their relationship with each other. To understand what the function of a particular distal region might be, genetics has long relied on reporter assays. Applicable mostly in model systems and cell lines, one candidate variant at a time is placed next to a reporter gene that enables the quantification of its influence on the expression of the reporter, such as a fluorescent protein. While allowing for *in vivo* insights, interpretation of reporters is limited in a number of ways: candidate variants **(i)** are typically tested outside of their native sequence and chromatin context, **(ii)** may comprise one, or only a part of a larger, regulatory region; and **(iii)** in the case of plasmid reporter constructs, are studied in the absence of chromatin.

To address the limited throughput, recent developments include massively parallel reporter assays that streamline and scale up the cloning of functional candidate fragments next to the reporter gene, allowing for readouts of thousands of fragments [32]. Placing short sequence fragments into the same controlled context holds promise for eliminating unwanted noise, thereby facilitating an unbiased evaluation of the impact of sequence variants. One example is **S**elf-**T**ranscribing **A**ctive **R**egulatory **R**egion sequencing (STARR-seq). This method is based on the knowledge that enhancers can work independently of their relative locations. Placing an enhancer candidate sequence downstream of a minimal promoter enables active enhancers to transcribe themselves, meaning that it can be read out and quantified by RNA-seq [33]. In spite of the limitations of these reporter assays, e. g., the differences observed when placing the same sequence within native chromatin or on plasmids [34], such data have laid the foundation for promising computational models that identify non-coding variants of clinical relevance [35].

To dissect the impact of a regulatory region within its native genomic context, one of the most promising directions has been opened by CRISPR genome editing tools. As is the case for protein coding genes, CRISPR editing can be targeted to specifically alter regulatory regions by means of complementary guide RNAs. As we often do not know which individual sequence features are relevant, saturation screens that introduce thousands of mutations help to comprehensively assay a larger genomic region, such as an entire TAD [36]. In pooled screens, the effect of a sequence or chromatin change is detected by phenotypes such as growth or proliferation [37]. In single-cell screens, the effect on gene expression is directly determined *via* single-cell RNA-seq [38].

Alternatively, “epigenome editing” perturbs the chromatin state *via* the introduction of inhibitory or activating marks (CRISPRi/a) [39]. In this approach, a dead Cas9 nuclease is fused to epigenome modifier domains, such as histone (de)acetylases, and is directed to a specific genomic location *via* a guide RNA [34]. As the whole regulatory region is affected instead of a small sequence, data acquisition scales better and interpretation is easier [40], [41].

## Single-cell genomics

With the requirements on sample size and purity decreasing, large consortia have made progress, moving from profiling of immortalized cell lines (ENCODE [11]) to *post mortem* fetal tissues (Roadmap [12]) and *post mortem* adult samples of healthy organs (GTEx consortium [42]). Genomics protocols have traditionally required tens of thousands to millions of cells to generate high-quality data. This meant that small cell populations, or short-lived states during development, were either not well reflected or entirely obscured in the averaged pictures resulting from these approaches. This situation has changed rapidly with the rise of single-cell genomics where sequencing protocols on individual cells [43] using microfluidic devices, liquid handling robotics, and/or clever multiplexed experimental designs enable us to profile tens of thousands and even millions of cells in a single experiment [44]. In contrast to the traditional, single average dataset generated from millions of cells and encompassing millions of short sequencing reads, each single-cell dataset is composed of a limited number of reads in the range of 10,000 to 100,000 reads. However, obtaining tens of thousands single-cell datasets from one experiment enables us to uncover heterogeneous states and responses of neighboring cells in a tissue [45].

Due to the highly parallel nature of these experiments, protocols that could be implemented in a few steps were adopted first, starting with RNA-seq for expression profiling. Yet other protocols, such as single-cell ATAC-seq, quickly followed and are now available as standardized kits from commercial vendors. Meanwhile, large atlases of gene expression and open chromatin in multiple systems have been generated for multiple organs [46], [47], [48]. Bisulfite sequencing and ChIP-seq are also implemented, but are not yet as widely adopted [44], [45]. With the possibility of obtaining high-resolution information from small, complex, and primary samples, single-cell genomics will transform basic genomics research and its clinical applications.

Since the start of genome sequencing projects, computational biology algorithms have been indispensable for organizing and interpreting today’s massive heterogeneous sources of data from bulk and single-cell experiments. In particular, carefully designed and vetted machine learning methods hold great promise for interpreting the complex rules of gene regulation that are spread out over large genomic regions and involve multiple mechanisms [35], [49]. As with other applications, a critical aspect of successful adaptation of this technique will be the ability to explain physiology and provide meaningful interpretations.

## Conclusion

High-throughput biology, and in particular deep sequencing, has made exploring the vast space of non-protein coding genomic regions possible. We now have a large catalog of candidate regulatory regions at our disposal, building the foundation that will enable us to include non-coding sequence variation into diagnostics and clinical practice in the context of the underlying causes of rare diseases.
